# Decision tree model for predicting long-term outcomes in children with out-of-hospital cardiac arrest: a nationwide, population-based observational study

**DOI:** 10.1186/cc13951

**Published:** 2014-06-27

**Authors:** Yoshikazu Goto, Tetsuo Maeda, Yumiko Nakatsu-Goto

**Affiliations:** 1 Section of Emergency Medicine, Kanazawa University Hospital, 13-1 Takaramachi, Kanazawa 920-8641, Japan; 2 Department of Cardiology, Yawata Medical Center, 12-7 I Yawata, Komatsu 923-8551, Japan

## Abstract

**Introduction:**

At hospital arrival, early prognostication for children after out-of-hospital cardiac arrest (OHCA) might help clinicians formulate strategies, particularly in the emergency department. In this study, we aimed to develop a simple and generally applicable bedside tool for predicting outcomes in children after cardiac arrest.

**Methods:**

We analyzed data of 5,379 children who had undergone OHCA. The data were extracted from a prospectively recorded, nationwide, Utstein-style Japanese database. The primary endpoint was survival with favorable neurological outcome (Cerebral Performance Category (CPC) scale categories 1 and 2) at 1 month after OHCA. We developed a decision tree prediction model by using data from a 2-year period (2008 to 2009, *n* = 3,693), and the data were validated using external data from 2010 (*n* = 1,686).

**Results:**

Recursive partitioning analysis for 11 predictors in the development cohort indicated that the best single predictor for CPC 1 and 2 at 1 month was the prehospital return of spontaneous circulation (ROSC). The next predictor for children with prehospital ROSC was an initial shockable rhythm. For children without prehospital ROSC, the next best predictor was a witnessed arrest. Use of a simple decision tree prediction model permitted stratification into four outcome prediction groups: good (prehospital ROSC and initial shockable rhythm), moderately good (prehospital ROSC and initial nonshockable rhythm), poor (prehospital non-ROSC and witnessed arrest) and very poor (prehospital non-ROSC and unwitnessed arrest). By using this model, we identified patient groups ranging from 0.2% to 66.2% for 1-month CPC 1 and 2 probabilities. The validated decision tree prediction model demonstrated a sensitivity of 69.7% (95% confidence interval (CI) = 58.7% to 78.9%), a specificity of 95.2% (95% CI = 94.1% to 96.2%) and an area under the receiver operating characteristic curve of 0.88 (95% CI = 0.87 to 0.90) for predicting 1-month CPC 1 and 2.

**Conclusions:**

With our decision tree prediction model using three prehospital variables (prehospital ROSC, initial shockable rhythm and witnessed arrest), children can be readily stratified into four groups after OHCA. This simple prediction model for evaluating children after OHCA may provide clinicians with a practical bedside tool for counseling families and making management decisions soon after patient arrival at the hospital.

## Introduction

Pediatric out-of-hospital cardiac arrest (OHCA) is an uncommon event, and outcomes following OHCA are considerably worse than in-hospital cardiac arrest [1-4]. Despite ongoing efforts to improve the quality of pediatric cardiopulmonary resuscitation (CPR), ≤10% of children with OHCA survive to hospital discharge and many have severe neurological sequelae [1,2,5].

Outcomes in children after OHCA depend on a multitude of variables, including age, specific diseases (for example, traumatic cardiac arrest and sudden infant death syndrome), initial recorded cardiac rhythm and other circumstances related to cardiac arrest, such as prolonged periods of no blood flow or day of arrests [1,2,5,6]. Prediction of outcome has the potential to help guide decision-making and risk assessment for individual patients [7]. An ideal prediction model for outcome may provide objective information about whether future support is likely to result in a good neurological outcome or survival. In adults with OHCA, multivariate analyses have identified factors that have enabled the development of sophisticated equations and scoring models, thus providing the ability to predict outcomes following cardiac arrest [8-11]. Although multiple clinical and physical examination findings, imaging, and electrographic features might be useful in predicting outcomes in children with OHCA [12,13], these prognostic indicators focus on the status of the patient after sustained return of spontaneous circulation (ROSC) or hospital admission following cardiac arrest. However, to our knowledge, an outcome prediction model for children with OHCA that an emergency department physician could apply soon after patient arrival at the hospital has not yet been developed. Furthermore, there have been discrepancies between pediatric emergency physicians and adult-oriented emergency physicians in treating children with unresponsive OHCA [14]. Therefore, a simple and reliable prediction model for all clinicians is required to counsel families and make management decisions in managing children with OHCA.

In this study, we aimed to establish and validate a new prediction model for emergency physicians treating OHCA children that would allow them to decide on in-hospital strategies immediately after patient arrival at the emergency department (ED).

## Material and methods

### Study design and data source

The present investigation was a nationwide, population-based observational study of all children (age <18 years) for whom resuscitation had been performed in Japan after OHCA between 1 January 2008 and 31 December 2010. “Cardiac arrest” was defined as the cessation of cardiac mechanical activity confirmed by the absence of signs of circulation [15]. The cause of arrest was presumed to be of cardiac origin, unless evidence suggested an external cause (trauma, hanging, drowning, drug overdose or asphyxia), respiratory disease, cerebrovascular disease, malignant tumor or any other noncardiac cause. The attribution of noncardiac or cardiac cause was made by the physicians in charge, in collaboration with the emergency medical services (EMS) personnel. We considered for analyses the cases of all children who received resuscitation, regardless of whether the causes of cardiac arrest were traumatic or not. This study was approved by the ethics committee of Kanazawa University. According to the informed consent guidelines in Japan [16], it is unnecessary to obtain informed consent from each patient to use secondary data from an anonymous database; therefore, this requirement for written informed consent was waived.

### Emergency medical services system in Japan

Japan has approximately 127 million residents in an area of 378,000 km^2^, approximately two-thirds of which is uninhabited mountainous terrain. Details of the Japanese EMS system have been described previously [8,17-19]. Briefly, municipal governments provide EMS through approximately 800 fire stations with dispatch centers. The Fire and Disaster Management Agency (FDMA) of Japan supervises the nationwide EMS system, and each local EMS system is operated by the local fire station. Generally, an ambulance crew includes three EMS staff members, including at least one emergency lifesaving technician (ELST). Under online medical control, ELSTs are allowed to use several resuscitation methods, including semiautomated external defibrillators, insertion of supraglottic airway devices (laryngeal mask airway, laryngeal tube and esophageal-tracheal twin-lumen airway device), insertion of peripheral intravenous lines and infusion of Ringer lactate solution. Since July 2004, only specially trained ELSTs are permitted to insert a tracheal tube, and, since April 2006, they have been permitted to administer intravenous epinephrine in the field, under the instruction of a physician online. Since October 2006, EMS providers perform CPR according to the Japanese CPR guidelines [20], which are based on the 2005 American Heart Association guidelines [21]. Because do not resuscitate orders and living wills are not generally accepted in Japan, and because EMS personnel in Japan are legally prohibited from terminating resuscitation in the field, most OHCA patients receive CPR from EMS providers and are transported to hospitals, except in specific situations (that is, decapitation, incineration, decomposition, rigor mortis or dependent cyanosis) [18,20]. The duration of on-scene effort by EMS personnel before transport is initiated is not predetermined.

### Data collection and quality control

The FDMA launches a prospective, population-based observational study that includes all OHCA patients who received EMS in Japan since January 2005 [2,8,15]. EMS personnel at each center record data from OHCA patients, with the cooperation of the physician in charge, using an Utstein-style template [22]. The data are transferred to their fire stations and are then integrated into the registry system on the FDMA database server. The data are checked for consistency by the computer system and are confirmed by FDMA personnel. If the data form is incomplete, the FDMA returns it to the respective fire station, where the form is completed [15]. All data are stored in the nationwide database developed by the FDMA for public use. The FDMA granted permission to analyze this database and provided all the anonymous data to our research group. The main variables included in the database are sex, age, cause of arrest (presumed cardiac etiology or not), bystander witness status, bystander CPR, use of automated external defibrillator (AED), initial identified cardiac rhythm, bystander category (that is, the presence or absence of a bystander or whether the bystander was a layperson or an EMS staff member), achievement of ROSC before arrival at the hospital, time of the emergency call, time of vehicle arrival at the scene, time of ROSC, time of vehicle arrival at the hospital, time of epinephrine administration, survival and neurological outcome at 1 month after cardiac arrest. The time data are recorded electronically using a recording device. The neurological outcome was defined according to the Cerebral Performance Category (CPC) scale: category 1, good cerebral performance; category 2, moderate cerebral disability; category 3, severe cerebral disability; category 4, coma or vegetative state; and category 5, death [22]. The CPC categorization was determined by the physicians in charge. The call-to-response time interval was calculated as the time from the emergency call to the time of vehicle arrival at the scene. The call-to-hospital arrival time interval was calculated as the time from the emergency call to the time of vehicle arrival at the hospital.

### Endpoints

The primary study endpoint was a 1-month favorable neurological outcome (defined as a CPC of 1 or 2) [22]. The secondary endpoint was survival at 1 month after OHCA.

### Statistical analyses

The Kolmogorov–Smirnov–Lilliefors test was performed to evaluate the distributions of continuous variables, by which we found a nonnormal distribution of all continuous variables (all *P* < 0.01). Therefore, the Wilcoxon and Kruskal–Wallis tests for continuous variables and the χ^2^ test for categorical variables were performed to compare the characteristics and outcomes between the cohorts. Multivariate logistic regression analyses including 11 variables were performed to assess the association between prehospital variables and 1-month survival or CPC category 1 or 2 at 1 month after OHCA in the group used for model development. We selected 11 variables obtained before hospitalization to develop a prediction model. These 11 variables, related to patient characteristics and resuscitation, were age, sex, witnessed arrest (yes or no), arrest witnessed by EMS personnel (yes or no), bystander CPR (yes or no), presumed cardiac etiology (yes or no), initial cardiac rhythm recorded (shockable or not), prehospital AED use (yes or no), call-to-response time interval, call-to-hospital arrival time interval and prehospital ROSC. Eight of the variables were considered dichotomous.

Because a recursive partitioning analysis might be a more suitable test than logistic regression when the intent is to classify one outcome at the expense of another [8,23], we performed a recursive partitioning analysis to develop a decision tree model to predict outcome. Recursive partitioning analysis creates a branching decision tree by dividing the patient population into subgroups according to the results of an analysis of the relationship between proportions of outcomes after OHCA and variables obtained prehospitalization. The recursive partitioning analysis was conducted using the maximized entropy index [24-26]. Tenfold cross-validation was used to assess the predictive ability of the decision tree model.

Wilcoxon and Kruskal–Wallis tests for continuous variables and χ^2^ tests for categorical variables were used. Continuous variables are expressed as medians (25th to 75th percentiles). Categorical variables are expressed as percentages. As an estimate of effect size and variability, we report odds ratios (ORs) with 95% confidence intervals (CIs). We assessed overall model discrimination by using sensitivity, specificity and the area under the receiver operating characteristic curve (AUC). All statistical analyses were performed with the JMP statistical package version 9 (SAS Institute, Cary, NC, USA). All tests were two-tailed, and a value of *P* < 0.05 was considered statistically significant.

## Results

**Figure 1:**
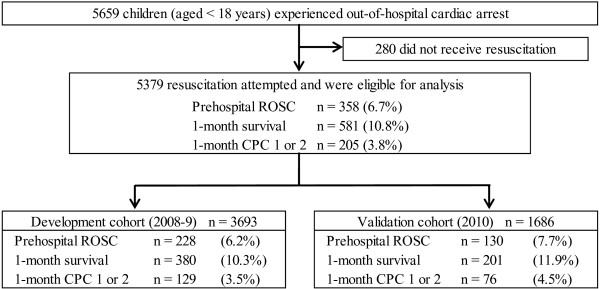
**Study profile showing flow diagram of participants.** CPC, Cerebral Performance Category; ROSC, Return of spontaneous circulation.

During the 3-year study period, 5,659 patients were documented in the database. We considered 5,379 patients (95.1%) eligible for enrollment into this study. Figure 1 is a flow diagram depicting the inclusion and exclusion criteria for subjects in the present study. The overall 1-month survival and favorable neurological outcomes (CPC category 1 or 2) were 10.8% and 3.8%, respectively. We developed a decision tree model by using data from a 2-year period (development cohort, 2008 and 2009, *n* = 3,693), which we validated using external data from 2010 (validation cohort, *n* = 1,686). The characteristics of all the subjects and the results of analyses between the two cohorts used for the development and validation of the models are shown in Table 1. Due to the large size of the study population, several significant differences were noted in baseline characteristics between the two cohorts: age (*P* < 0.0001), bystander CPR (*P* = 0.027), presumed cardiac etiology (*P* = 0.001), prehospital AED administration (*P* = 0.036), call-to-response time (*P* = 0.006) and call-to-hospital arrival time (*P* = 0.025). However, sizable differences were not frequent, except for the proportion of patients age <1 year. Although the validation cohort had a significantly higher prehospital ROSC rate than the development cohort (*P* = 0.039), there were no significant differences between the two cohorts in 1-month survival or 1-month CPC 1and 2 rates (survival, *P* = 0.079; CPC 1 and 2, *P* = 0.077).

**Table 1:** **Baseline characteristics and outcomes of the study patients**^**a**^

**Characteristics**	**All patients**	**Development cohort (2008 and 2009)**	**Validation cohort (2010)**
Number of patients	5,379 (100%)	3,693 (68.7%)	1,686 (31.3%)
Age, yr	1 (0 to 11)	1 (0 to 10)	2 (0 to 13)
<1 yr^b^	2,328 (43.3%)	1,713 (46.4%)	615 (36.5%)
Boys	3,261 (60.6%)	2,222 (60.2%)	1,039 (61.6%)
Witnessed arrest	1,764 (32.8%)	1,182 (32.0%)	582 (34.5%)
Family member	835 (15.5%)	554 (15.0%)	281 (16.7%)
EMS personnel	370 (6.9%)	246 (6.7%)	124 (7.4%)
Bystander CPR^c^	2,740 (50.9%)	1,845 (50.0%)	895 (53.1%)
Presumed cardiac etiology^d^	1,792 (33.3%)	1,283 (34.7%)	509 (30.2%)
Shockable initial cardiac rhythm	261 (4.9%)	192 (5.2%)	69 (4.1%)
Prehospital AED administration^e^	315 (5.9%)	233 (6.3%)	82 (4.9%)
Call-to-response time (min), *n* = 5,360 (99.6%)^f^	7 (5 to 8)	7 (5 to 8)	7 (5 to 9)
Call-to-hospital arrival time (min), *n* = 5,326 (99.0%)^g^	27 (21 to 35)	27 (21 to 35)	28 (21 to 37)
Prehospital ROSC	358 (6.7%)	228 (6.2%)	130 (7.7%)
1-month outcome after cardiac arrest			
Survival	581 (10.8%)	380 (10.3%)	201 (11.9%)
Favorable neurological outcome (CPC = 1 or 2)	205 (3.8%)	129 (3.5%)	76 (4.5%)

^a^AED, automated external defibrillator; CPC, Cerebral Performance Category; CPR, Cardiopulmonary resuscitation; EMS, Emergency medical services; ROSC, Return of spontaneous circulation. ^b^*P* < 0.0001. ^c^*P* = 0.027. ^d^*P* = 0.001. ^e^*P* = 0.036. ^f^*P* = 0.006. ^g^*P* = 0.025. Values are reported as either number of patients (%) or median (25th to 75th percentiles).

Table 2 shows the results of multivariate logistic regression analyses for prehospital variables associated with 1-month outcomes. Five variables were independently associated with increased odds of both 1-month survival and 1-month CPC 1 or 2: witnessed arrest, shockable initial rhythm, presumed cardiac etiology, bystander CPR and prehospital ROSC. Of those, prehospital ROSC was the strongest predictive factor, with ORs of 22.9 (95% CI = 17.4 to 30.3) and 39.3 (95% CI = 27.0 to 58.2) for 1-month survival and 1-month CPC 1 or 2, respectively.

**Table 2:** **Results of multivariate logistic regression analyses for prehospital variables associated with 1-month outcomes**^**a**^

**Characteristics**	**Adjusted OR (95% CI)**	
	**1-month survival**	**1-month CPC 1 or 2**
Age,^b^ yr	0.96 (0.94 to 0.98)	0.98 (0.95 to 1.01)
Boys	0.86 (0.70 to 1.05)	1.04 (0.72 to 1.49)
Witnessed arrest	1.90 (1.51 to 2.37)	3.82 (2.54 to 5.82)
Arrest witnessed by EMS personnel	0.89 (0.59 to 1.34)	1.39 (0.77 to 2.46)
Shockable initial rhythm	1.90 (1.16 to 3.10)	2.38 (1.14 to 4.93)
Prehospital AED administration	2.14 (1.32 to 3.41)	1.87 (0.88 to 3.84)
Presumed cardiac etiology	1.33 (1.06 to 1.67)	1.49 (1.00 to 2.22)
Bystander CPR	1.48 (1.19 to 1.84)	1.52 (1.03 to 2.28)
Call-to-response time^b^ (min)	1.00 (0.96 to 1.03)	1.05 (0.97 to 1.11)
Call-to-hospital arrival time^b^ (min)	0.99 (0.98 to 0.99)	0.98 (0.97 to 0.99)
Prehospital ROSC	22.9 (17.4 to 30.3)	39.3 (27.0 to 58.2)

^a^AED, Automated external defibrillator; CI, Confidence interval; CPC, Cerebral Performance Category; CPR, Cardiopulmonary resuscitation; EMS, Emergency medical services; OR, Odds ratio; ROSC, Return of spontaneous circulation. ^b^Adjusted OR is reported for unit odds.

**Figure 2:**
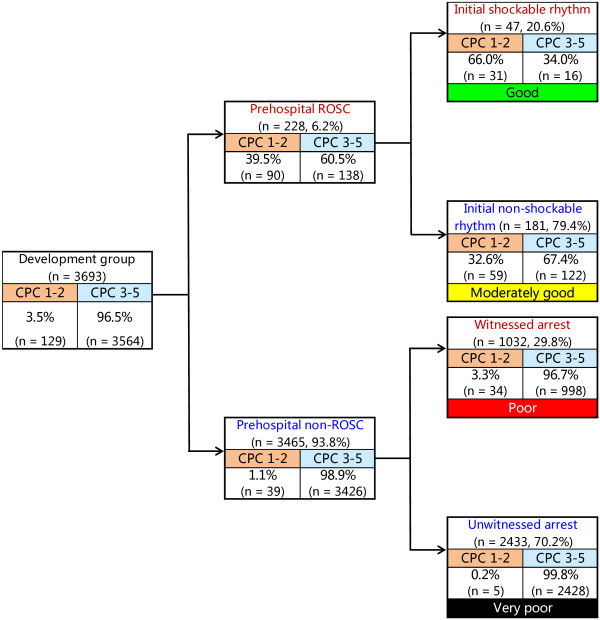
**Decision tree model of recursive partitioning analysis for predicting favorable neurological outcomes at 1 month and prediction groups in the model development cohort.** CPC, Cerebral Performance Category; ROSC, Return of spontaneous circulation.

**Figure 3:**
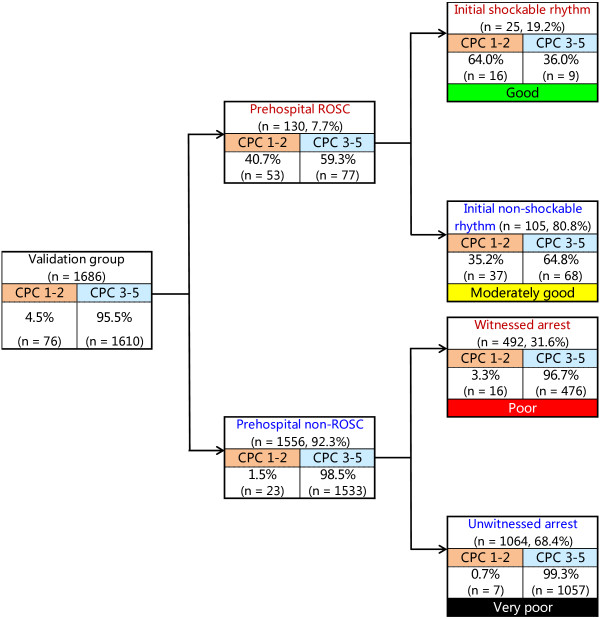
**Decision tree model of recursive partitioning analysis for predicting favorable neurological outcomes at 1 month and prediction groups in the validation cohort.** CPC, Cerebral Performance Category; ROSC, Return of spontaneous circulation.

**Figure 4:**
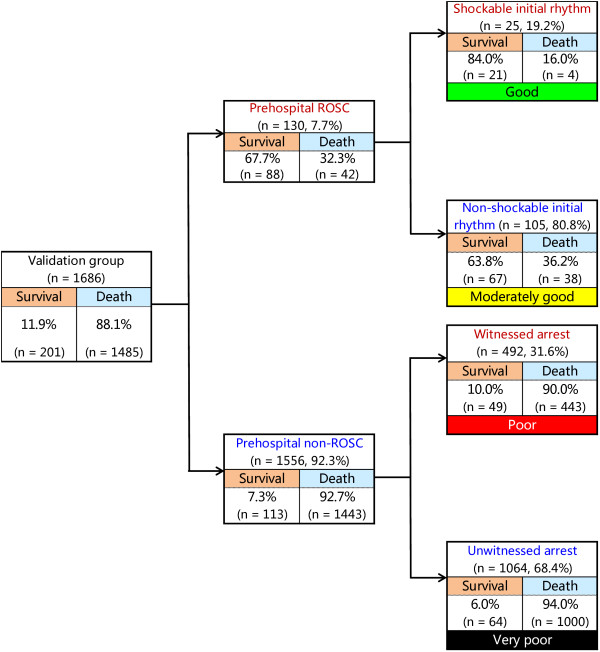
**Decision tree model of recursive partitioning analysis for predicting survival at 1 month and prediction groups in the validation cohort.** CPC, Cerebral Performance Category; ROSC, Return of spontaneous circulation.

Figure 2 depicts the decision tree model of the recursive partitioning analysis for predicting favorable neurological outcome at 1 month in the development cohort. In the analysis, we identified prehospital ROSC as the best single discriminating factor between categories CPC 1 and 2 and CPC 3 to 5. The next best predictor of neurological outcome in the prehospital ROSC node was initial shockable rhythm. In the prehospital non-ROSC node, the next best predictor of neurological outcome was witnessed arrest. These branch points permitted stratification into four prediction groups: good (prehospital ROSC and initial shockable rhythm), moderately good (prehospital ROSC and initial nonshockable rhythm), poor (prehospital non-ROSC and witnessed arrest) and very poor (prehospital non-ROSC and unwitnessed arrest). The prediction values of CPC 1 and 2 ranged from 0.2% to 66.0% in the very poor and good groups. The decision tree model generated by the recursive partitioning analysis was tested for its ability to stratify patients using the validation cohort (Figure 3). The AUCs of this model for predicting CPC 1 and 2 in the model development and validation cohorts were 0.92 (95% CI = 0.91 to 0.92) and 0.88 (95% CI = 0.87 to 0.90), respectively. The validated decision tree prediction model showed a sensitivity of 69.7% (95% CI = 58.7% to 78.9%) and a specificity of 95.2% (95% CI = 94.1% to 96.2%) for predicting 1-month CPC 1 and 2. In addition, the decision tree generated by analysis of the development cohort for 1-month CPC 1 and 2 was tested for its ability to stratify patients for 1-month survival using the validation cohort (Figure 4). The prediction values for survival ranged from 6.0% to 84.0% in the very poor and good groups. The AUCs of this model for predicting 1-month survival in the cohorts of development and validation were 0.73 (95% CI = 0.71 to 0.75) and 0.74 (95% CI = 0.71 to 0.77), respectively. The validated decision tree prediction model showed a sensitivity of 68.2% (95% CI = 61.4% to 74.2%) and a specificity of 67.5% (95% CI = 65.1% to 69.9%) for predicting 1-month survival. Table 3 summarizes the definition of prediction groups for children with OHCA using three prehospital factors.

**Table 3:** **Definition of prediction groups for children after out-of-hospital cardiac arrest**^**a**^

**Prehospital factors**	**Prediction groups**			
	**Good**	**Moderately good**	**Poor**	**Very poor**
Prehospital ROSC	Yes	Yes	No	No
Shockable initial rhythm	Yes	No		
Witnessed arrest			Yes	No

^a^ROSC, Return of spontaneous circulation.

## Discussion

In the present analysis of more than 5,000 Japanese children who experienced OHCA, we demonstrate that, by using the appropriate AUC values, neurological outcomes and survival at 1 month after cardiac arrest can be reliably estimated in the ED based on three routinely available prehospital variables: prehospital ROSC, initial shockable rhythm and witnessed arrest. This prediction model of a decision tree for children after OHCA might provide emergency physicians with a practical bedside tool for counseling families and making medical decisions in the ED.

Michiels *et al*. [27] recently demonstrated that long-term survival for at least 10 years following hospital discharge after OHCA was generally favorable for all children who were discharged with a favorable neurological outcome. Their findings suggest that functional status at the time of hospital discharge can be used to make a meaningful prediction of long-term survival. Therefore, we considered our primary outcome, namely, 1-month favorable neurological outcome, a surrogate for long-term survival in children after OHCA.

In the present study, five variables, determined by the multivariate logistic analysis, were significantly associated with increased odd of favorable neurological outcome: prehospital ROSC, witnessed arrest, shockable initial rhythm, bystander CPR and presumed cardiac etiology (Table 2). Of these, the most crucial prehospital factor for predicting 1-month outcome was prehospital ROSC. The factor prehospital ROSC had the highest adjusted OR for both 1-month survival and 1-month CPC 1 or 2 (Table 2). This finding is consistent with that in a previous study by Sasson *et al*. [28]. They demonstrated that the most powerful predictor associated with survival after OHCA in adults was prehospital ROSC, irrespective of the subsequent sophistication level of in-hospital care. The second crucial prehospital factor we evaluated was witnessed arrest. Although no predictors of out-of-hospital resuscitation success or failure have been established in children with OHCA [29,30], Atkins *et al*. [1] demonstrated that only age group (1 to 20 years) and witnessed arrest were significantly associated with survival. Kitamura *et al*. [2] reported that age group (1 to 17 years), witnessed arrest, initial ventricular fibrillation, any bystander CPR and earlier initiation of CPR by EMS personnel were significantly associated with improved neurological outcome after OHCA in children. However, Mole *et al*. [6] observed that age was not associated with survival when tested either as a continuous variable or as an Utstein-style age category. In the present study, age, used as a continuous variable, was associated with a decreased OR of 1-month survival, but there was no association between age and 1-month neurological outcome (Table 2). The third crucial prehospital factor we examined was shockable rhythm. In previous studies, researchers have shown that an initial shockable rhythm was associated with achievement of sustained ROSC [31], survival to hospital discharge [32] and favorable 1-month neurological outcomes in children with OHCA [2,33]. Therefore, the present results support those of previous studies. On the basis of our recursive partitioning analyses, we included these three factors in the decision tree model for predicting outcome in children after OHCA.

The 2010 American Heart Association Guidelines for Cardiopulmonary Resuscitation and Emergency Cardiovascular Care [29] provide two termination of resuscitation (TOR) criteria to be used by EMS personnel to predict survival after OHCA in adults. These rules include five prehospital variables: incident witnessed by a bystander, incident witnessed by EMS personnel, bystander CPR, initial shockable rhythm and ROSC in the field. Sasson *et al*. [28] demonstrated the value of bystander CPR, the critical importance of shockable rhythm and the predictive value of ROSC in the prehospital setting in their meta-analysis of adults after OHCA. They also showed that the magnitude of effect sizes for these five clinical factors is higher in communities that have lower baseline survival rates. This means that the crucial predictors of outcomes after OHCA differ according to the EMS system and baseline survival rates in a region. Among the five predictors in the guideline for OHCA in adults [29] evaluated in the present study, the only prehospital variable that was not included as an independent predictor for OHCA children was EMS-witnessed arrest (Table 2).

In Japan, Goto *et al*. [18] demonstrated that in adults after OHCA, three prehospital factors were associated with increased ORs of 1-month survival and 1-month neurological outcomes: prehospital ROSC, initial shockable rhythm and bystander witness. In the present study aimed at predicting outcomes in children after OHCA, we, too, found that the same three prehospital factors were crucial predictors of 1-month outcomes by using recursive partitioning analyses. Although the causes of cardiac arrest in children are different from those in adults, such as sudden infant death syndrome, respiratory distress and asphyxia, they are of great importance in predicting outcomes after OHCA. Conceivably, the prehospital predictors for adults after OHCA might be applicable to children after OHCA in the same region by taking into consideration potential sources of variation such as the type of EMS system and baseline survival rates.

In recent years, TOR in the out-of-hospital setting has become more widespread in adults after OHCA following futile resuscitation [29]. Although TOR by EMS personnel in the field is not generally accepted in children after OHCA [29,30], several EMS systems have had experience using TOR procedures for children [34]. However, EMS personnel in Japan are not allowed to implement TOR in the field [18,19]. A TOR rule for physicians treating adults in the ED after OHCA has recently been developed and validated [18]. Previously, Scribano *et al*. demonstrated that general emergency physicians were less likely than pediatric emergency physicians to halt futile resuscitation in pediatric patients [14]. This finding may reflect the notion that poor knowledge of the literature on pediatric outcome or less training or skills confidence in treating children contributes to this attitude [34]. Our decision tree model, which includes only three prehospital variables, is a very simple and easily accessible model for any physician in the ED to predict outcomes of children after OHCA. If a child with unwitnessed OHCA is transported to the hospital before ROSC, even an adult-oriented physician can immediately understand that the child will have a very poor outcome (Table 3) and can counsel the family on whether to terminate the futile resuscitation. On the contrary, if a child has prehospital ROSC together with an initial shockable rhythm, the physician can immediately expect survival with a favorable neurological outcome (Table 3) and should perform resuscitation with advanced life support according to accepted guidelines [35,36]. Collectively, our prediction model for children after OHCA might provide all clinicians with a practical bedside tool for counseling families and making management decisions soon after patient arrival at the ED.

New treatments for cardiac arrest and improvements in prehospital system factors may improve outcomes following OHCA. To carry a self-fulfilling prophecy in implementing our prediction model, it should be challenged periodically as new treatments emerge or social systems evolve.

### Study limitations

The potential limitations of the current analysis are as follows. First, our database lacked detailed data with which to make further risk adjustments for outcomes, such as comorbid diseases, location of OHCA occurrence, quality of EMS personnel, degree of regional differences among EMS centers, in-hospital medications and the availability emergency care specialists. These data were not evaluated, owing to the retrospective design of our study. Second, although we used uniform data collection procedures based on the Utstein-style guidelines for reporting cardiac arrest and the study had a large sample size and a population-based design, we cannot exclude the possibility of the presence of uncontrolled confounders. Third, as with all epidemiological studies, the integrity, validity and ascertainment bias of the data are potential limitations. Fourth, the generalizability of our results to other countries could be limited because the data we analyzed were derived from only a Japanese nationwide population-based cohort of OHCA patients in Japan. Therefore, it may be necessary for researchers to validate the present prediction model in other countries. Finally, as we did not have precise data concerning the causes of cardiac arrest, there is a possibility that some of patients may have had sudden infant death syndrome.

## Conclusions

On the basis of our decision tree prediction model with three prehospital variables (prehospital ROSC, initial shockable rhythm and witnessed arrest), children can be readily stratified into four groups after OHCA (good, moderately good, poor and very poor) that can help predict both 1-month survival and 1-month favorable neurological outcome. This prediction model demonstrated a sensitivity of 69.7%, a specificity of 95.2% and an AUC of 0.88 for predicting 1-month favorable neurological outcome in our validation study. This simple prediction model may provide clinicians with a practical bedside tool for counseling families and making management decisions soon after pediatric patient arrival in the ED.

## Key messages

• We have developed and validated a simple and generally applicable decision tree prediction model for children in the ED after OHCA by using a prospectively recorded, nationwide, Utstein-style Japanese database.

• The decision tree model consists of three prehospital variables: prehospital ROSC, initial shockable rhythm and witnessed arrest.

• This model can readily stratify recovery in children after OHCA into groups of good, moderately good, poor and very poor prognosis by predicting a 1-month favorable neurological outcome and might help guide clinicians’ decision-making and risk assessment in these children.

## Abbreviations

AED: Automated external defibrillator; AUC: Area under the receiver operating characteristic curve; CI: Confidence interval; CPC Scale: Cerebral Performance Categories Scale; CPR: Cardiopulmonary resuscitation; ED: Emergency department; ELST: Emergency lifesaving technician; EMS: Emergency medical services; FDMA: Fire and Disaster Management Agency; OHCA: Out-of-hospital cardiac arrest; OR: Odds ratio; ROSC: Return of spontaneous circulation; TOR: Termination of resuscitation.

## Competing interests

The authors declare that they have no competing interests.

## Authors’ contributions

YG and TM designed the study. YG, TM and YN conducted data cleaning. YG and YN analyzed the data. YG drafted the manuscript, and YN and TM contributed substantially to its revision. YG takes responsibility for the article as a whole. All authors read and approved the final manuscript.
